# Regulation of tight junction gene expression in the kidney of calbindin-D9k and/or -D28k knockout mice after consumption of a calcium- or a calcium/vitamin D-deficient diet

**DOI:** 10.1186/1471-2091-15-6

**Published:** 2014-03-15

**Authors:** Inho Hwang, Eui-Ju Hong, Hyun Yang, Hong-Seok Kang, Changhwan Ahn, Beum-Soo An, Eui-Bae Jeung

**Affiliations:** 1 Laboratory of Veterinary Biochemistry and Molecular Biology, College of Veterinary Medicine, Chungbuk National University, Cheongju, Chungbuk 361-763, Republic of Korea; 2 Department of Biomaterials Science, College of National Resources & Life Science, Pusan National University, Busan, Republic of Korea

**Keywords:** Tight junction, Paracellular calcium transport, Calbindin

## Abstract

**Background:**

Calciotropic hormones were thought to facilitate calcium transfer through active transcellular or passive paracellular pathway for calcium homeostasis. While calcium transport proteins such as CaBP-28 k, TRPV5, NCX1, PMCA1b are involved in calcium reabsorption of the renal tubule using transcellular transport, tight junction proteins are known as critically related to calcium absorption through paracellular pathway. The regulation of each pathway for calcium transport was well studied but the correlation was not. It is expected that present study will provide new information about the link between transcellular and paracellular pathway within renal tubules.

**Results:**

Transcripts and proteins of tight junction related genes (occludin, ZO-1, and claudins) were examined in CaBP-9 k-and/or-28 k-deficient mice as well as the effect of dietary calcium and/or vitamin D supplementation. With a normal diet, the transcriptional and translational expressions of most tight junction proteins in the kidney was not significantly changed but with a calcium- and vitamin D-deficient diet, and they were significantly increased in the kidney of the CaBP-28 k and CaBP-9 k/28 k double KO (DKO) mice. In these genotypes, the increase of tight junction related transcripts and proteins are referred to as an evidence explaining correlation between transcellular transport and paracellular pathway.

**Conclusions:**

These findings are particularly interesting in evidences that insufficient transcellular calcium transports are compensated by paracellular pathway in calcium or calcium/vitamin D deficient condition, and that both transcellular and paracellular pathways functionally cooperate for calcium reabsorption in the kidney.

## Background

Calcium homeostasis is essential to maintain balance, which is required for the nerve impulses, muscle contraction, blood coagulation, cardiac functions, and other processes. Calcium imbalance may lead to various diseases such as renal stones, stroke and cardiovascular disease. Plasma calcium is reserved or supplied from bones through hormonal effect such as parathyroid hormone (PTH) and calcitonin. Calcium absorption or excretions occur through active transcellular- and passive paracellular pathways in the epithelia of intestine or kidney [1,2]. Renal calcium re-absorption is essential for maintaining blood calcium homeostasis, and which is mostly in the distal tubules and passively in the thick ascending limb of Henle’s loop (TAL).

Transcellular transports of calcium transport are performed by cooperation of calcium transport genes such as transient receptor potential cation channel subfamily V members (TRPVs) 5 and 6, sodium-calcium exchanger (NCX) 1, plasma membrane calcium ATPase-1b (PMCA1b), and calbindin-D9k/-D28k (CaBP-9 k/28 k). TRPV5, CaBP-28 k, NCX1, and PMCA1b are mainly acts for re-absorption of calcium [3]. TRPV5 facilitates intake of filtrated luminal calcium ion into the renal cell [4-6]. The entered calcium ion, bind with CaBP-28 k, and freely moving cellular space. CaBP-28 k helps to buffer intracellular calcium levels in the kidney, respectively [7,8]. In the external cell membrane, NCX1 and PMCA1b excrete intracellular calcium ion to blood. NCX1 accept outer cellular sodium ions and excretes inner cellular calcium ions, and PMCA1b facilitates the excretion of cell calcium ions [9-11].

The paracellular calcium re-absorption is occurred through intercellular tight junction proteins [12]. Among tight junction proteins, Myosin light chain kinase (MLCK), ZO-1, occludin (OCLN), and claudin (CLDN) families are known as involved in paracellular calcium transport. Myosin light chain kinase (MLCK) regulates the paracellular permeability, by inducing contraction of the perijunctional actomyosin ring through phosphorylation of myosin II regulatory light chain [13]. MLCK was known as regulator of intestinal tight junction permeability and involved in coordinating glucose transport and paracellular glucose permeability [14,15]. OCLN and ZO-1 are related to whole paracellular permeability. As a transmembrane protein, OCLN seals intercellular junctions therefore it makes dense the intercellular space [16,17]. ZO-1 is cytoplasmic plaque protein, which supply binding domain ‘PSD-95/Dlg/ZO-1 (PDZ)’ for transmembrane proteins [18,19]. Enhanced expression of OCLN and ZO-1 may increase transepithelial resistance (TER) that means bidirectional resistance through the intercellular junction.

CLDN families are transmembrane proteins. Especially, some CLDNs are known as have charge-selectivity [20]. Therefore, some charge-selective CLDNs are involved to calcium transport. Non charge-selective CLDNs also could affect to paracellular calcium transport by sealing intercellular junctions or by interacting with other charge-selective CLDNs. In the kidney, many studies have revealed that the interaction between CLDN16 with CLDN19 is critical for calcium and magnesium transport in the thick ascending limbs (TAL) of Henle’s loop [21-24]. CLDN14 is known as regulator of calcium reabsorption in response to calcium sensing receptor (CaSR) signaling [25]. CLDN10b is also reported to be crucial for calcium re-absorption in the TAL [26]. Other CLDNs such as CLDN1 and CLDN5 have been shown to have clear sealing functions that may also affect calcium transport because they influence general paracellular permeability [27,28]. CLDN4 which is mainly expressed in thin ascending limb of Henle’s loop and collecting duct is known as chloride channel, and it also has been reported as related to paracellular permeability. Overexpression of CLDN4 was induced increased TER through selective decrease of sodium ion permeability without chloride ion permeability [29].

Although transcellular transport or paracellular pathway has been well studied in calcium reabsorption in renal tubule [30], however, the interactions between these two calcium transports were not well organized. To investigate the correlation between transcellular and paracellular pathways in the kidney of mice, the expression and histologic alteration of tight junction proteins were evaluated in the situation of insufficient transcellular transport due to ablations of CaBP-9 k or/and CaBP-28 k. We also investigated the effect of insufficient dietary calcium or calcium/vitamin D to tight junction genes regulatory expression in the kidney of CaBP-9 k or/and -28 k knockout mice.

## Methods

### Animals

3 weeks old male wild-type literature (WT; C57BL/6), CaBP-9 k KO, CaBP-28 k KO, and CaBP-9 k/-28 k DKO mice were used. Mice lacking CaBP-9 k and/or -28 k gene were generated and the genotypes of the offspring were determined as previously described [31]. A total of 60 animals were divided into 12 groups (n = five per group) according to genotype and diet-type.

### Experimental treatments

To investigate the dietary effects of calcium and vitamin D, the mice were fed a normal diet (DYET #113295, AIN-76A purified rodent diet containing 0.8% phosphorus and 1.1% calcium; Dyets Inc., Bethlehem, PA, USA), a calcium deficient diet (DYET #113294, AIN-76A purified rodent diet with 1% phosphorous and 0.02% calcium, Dyets Inc.), or a calcium-/vitamin D-deficient diet (D10373A, AIN-76A-based diet containing 0.8% strontium, 0.02% calcium and 0.35% phosphorus; Research Diets, Inc., Brunswick, NJ, USA). All animals were fed the normal or experimental diets for 4 weeks (when the mice were 3 to 7 weeks old). All the mice were then euthanized with ether, and tissue samples from the kidney were collected. All animal experimental procedures were approved by the Ethics Committee of Chungbuk National University in Republic of Korea.

### Quantitative real-time PCR

Total RNA was extracted using TRIzol reagent (Ambion, Austin, TX, USA) according to the manufacturer’s instructions. The total RNA concentration was measured at 260 nm with EPOCH micro-volume spectrophotometer (BioTeK, Winooski, Vermont, USA). First strand complement DNA (cDNA) was synthesized by reverse transcription from 1 μg of total RNA using moloney murine leukemia virus (MMLV) reverse transcriptase (Invitrogen Co., Carlsbad, CA, USA) and random primers (9-mers; TaKaRa Bio Inc., Otsu, Shiga, Japan). Reverse transcription (RT) PCR was performed with a 7300 Real-Time PCR system (Applied Biosystems, Foster City, CA, USA) according to the manufacturer’s instruction. β-actin was used as an internal control for normalization and the relative gene expression levels were quantified using RQ software (Applied Biosystems).

Quantitative real-time PCR was performed for reactions containing 1 μL of cDNA template with 10 pmol of primers specific for tight junction genes and 10 μL of 2 x SYBR Premix ExTaq (TaKaRa Bio Inc.). The primer sequences are listed in Table 1. Quantitative real-time PCR was carried out for 40 cycles of denaturation at 95°C for 15 seconds (s), annealing at 62°C for 15 s, and extension at 72°C for 30s using an ABI Prism 7300 Sequence 10 detection system (Life Technologies). All mRNA values were monitored for an amplification curve, calculated based on the cycle threshold, and analyzed by universal 2 ΔΔCT method. The threshold cycle (Ct) was defined as the cycle when sample fluorescence reached the threshold level.

**Table 1:** List of primers used for PCR in this study

	**Forward**	**Reverse**
**β-actin**	5*'*-ACAGGCATTGTGATGGACTC-3*'*	5*'*-ATTTCCCTCTCAGCTGTGGT-3*'*
**OCLN**	5*'*-ACTGGGTCAGGGAATATCCA-3	5*'*-TCAGCAGCAGCCATGTACTC-3*'*
**ZO-1**	5*'*-ACTCCCACTTCCCCAAAAAC-3*'*	5*'*-CCACAGCTGAAGGACTCACA-3*'*
**CLDN1**	5*'*-AGGTCTGGCGACATTAGTGG-3*'*	5*'*-CGTGGTGTTGGGTAAGAGGT-3*'*
**CLDN4**	5*'*-ATCGTTGTCCGCGAGTTCTA-3*'*	5*'*-GCTTGTCGTTGCTACGAGGT-3*'*
**CLDN5**	5*'*-GCTGGTGGCACTCTTTGTTA-3*'*	5*'*-GCACCGTCGGATCATAGAAC-3*'*
**CLDN10b**	5*'*-TCGCCTTCGTAGTCTCCATC-3*'*	5*'*-TCTCCTTCTCCGCCTTGATAC-3*'*
**CLDN16**	5*'*-CTTGGCCATATTCTCCACTG-3*'*	5*'*-GAGTCGTACTCATCGCAGGT-3*'*
**CLDN19**	5*'*-TCCTCTTGGCAGGTCTCTGT-3*'*	5*'*-GTGCAGCAGAGAAAGGAACC-3*'*

### Western blot analysis

Proteins were extracted with PRO-PREP (iNtRON Biotechnology, Gyeoggi-Do, Republic of Korea) and homogenized. The protein samples were centrifuged at 14000 rpm, separated in 7.5 ~ 12.5% SDS-PAGE gels (40 μg per lane), and transferred to nitrocellulose membranes (Millipore, Bedford, MA, USA). The membranes were blocked with 5% skim milk in Tris-buffered saline with 0.5% Tween-20 (TBS-T) for 2 h at room temperature, and then incubated overnight (O/N) at 4°C with the following primary antibodies: anti-CLDN4 (1:1000, Invitrogen Co.), anti-CLDN16 (1:500, Invitrogen Co.), anti-β-actin (1:1000, Santa Cruz Biotechnology, Dallas, Texas, USA), mouse anti-CLDN1 (1:1000, Invitrogen Co.). Next, the membranes were washed with TBS-T for 1 h at room temperature, and incubated with anti-rabbit and anti-mouse horseradish peroxidase-conjugated secondary antibodies (1:3000, Santa Cruz Inc.) for 2 hours at room temperature. After subsequently washing the membranes with TBS-T, antibody binding was detected with an enhanced chemiluminescence reagent (Amersham Biosciences, Little Chalfont, UK) and detected by chemi doc equipment GenGnome 5 (Syngene, Cambridge, UK). To ensure signal specificity, the membranes were incubated with the secondary antibody alone. Density measurements for each band were performed with NIH Image J software. Background samples from an area near each lane were subtracted from each band to obtain mean band density.

### Immunohistochemistry

Histologic alteration of tight junction proteins was examined using immunohistochemistry. Samples of the kidney was embedded in paraffin, cut into 5- μm sections, deparaffinized with xylene, and hydrated in descending graded ethanol solutions. The sections were then mounted onto glass slides (Matunami, Ishikawa, Japan). Endogenous peroxidase activity was blocked by 3% hydrogen peroxidase in PBS for 30 minutes at room temperature. To prevent non-specific reactions, the sections were incubated with 10% goat serum (Vector Laboratories, Burlingame, CA, USA) in PBS for 1 hour at room temperature. After washing with TBS-T, the sections were incubated O/N at room temperature with the same primary antibodies used for Western blotting (rabbit anti-CLDN4 and - CLDN16; and mouse anti-CLDN1) diluted 1:250 with 5% BSA. The slides were washed with TBS-T before being incubated with biotinylated secondary antibodies (1:500, rabbit or mouse IgG; Vector Laboratories, Inc.) for 1 hour at 37°C and then ABC Elite solution (Vector Laboratories, Inc.) for 30 min at 37°C. Diaminobezidine (Sigma, St. Louis, MO, USA) was used as a chromogen. The sections were counterstained with hematoxylin and mounted in Cytoseal*60 (Richard-Allan Scientific Co., Kalamazoo, MI, USA).

### Data analysis

Data were analyzed with a nonparametric one-way analysis of variance (ANOVA) followed by *Tukey’s test* for multiple comparisons. All experiments were run of three separate experiments. All statistical analyses were performed using SPSS for Windows (SPSS, Chicago, IL, USA).

## Results

### Expression of tight junction genes in the kidney

To examine whether the change of tight junction related transcripts in the kidney is due to calcium or vitamin D deficient diet, we conducted a separate set of experiments in which the mRNA expressions of tight junction genes were measured and presented in Table 2. The median value of five sample replicates was used to calculate differentially expressed genes. Expression patterns of these genes varied although most appeared to be up-regulated. Significant regulation of OCLN expression was not detected in the kidney. When the calcium/vitamin D-deficient diet was administered, ZO-1 mRNA was up-regulated in CaBP-28 k KO and DKO mice compared to WT mice. The expression of ZO-1 was also higher in calcium-deficient DKO mice than WT animals fed the same diet. CLDN1 mRNA levels were higher in calcium-deficient CaBP-9 k KO and DKO mice than WT mice. CLDN4 mRNA of DKO mice were increased compared to WT mice regardless type of diets. In addition, CLDN4 mRNA of CaBP-28 k KO mice was up-regulated in calcium and calcium/vitamin D deficient. CLDN5 mRNA expression in the CaBP-28 k KO mice was increased with the calcium/vitamin D-deficient diet compared to the normal diet. CLDN5 mRNA levels were higher in calcium- and calcium/vitamin D-deficient DKO mice compared to the WT animals. These levels were also increased in the calcium/vitamin D-deficient CaBP-28 k KO mice relative to the corresponding WT controls. CLDN10b expression in the CaBP-28 k KO groups was up-regulated with the calcium- and calcium/vitamin D-deficient diets compared to the normal diet. Additionally, CLDN10b mRNA levels were higher in the calcium- and calcium/vitamin D-deficient CaBP-28 k KO mice than the WT animals. When the calcium-deficient diet was administered, CLDN16 mRNA was up-regulated in CaBP-9 k KO and DKO mice compared to WT mice. The level of CLDN16 mRNA in calcium/vitamin D-deficient diets was also increased in the calcium/vitamin D-deficient CaBP-28 k KO and DKO mice. Tight regulation of CLDN19 expression according to genotype and diet was not observed in the kidney.

**Table 2:** Tight junction gene regulation in the kidney

	**OCLN**	**ZO-1**	**CLDN1**	**CLDN4**	**CLDN5**	**CLDN10b**	**CLDN16**	**CLDN19**
Normal	-	-	-	DKO↑	-	-	-	-
Ca^2+^ deficient	-	DKO↑	9 k↑, DKO↑	28 k↑, DKO↑	DKO↑	28 k↑	9 k↑, DKO↑	-
Ca^2+^/Vit.D deficient	-	28 k↑, DKO↑	-	28 k↑ DKO↑	28 k↑ DKO↑	28 k↑	28 k↑, DKO↑	-

↑: Significantly higher than WT mice, -: no difference, 9 k: CaBP-9 k KO mice, 28 k: CaBP-28 k KO mice, DKO: CaBP-9 k and -28 k double-KO mice.

**Figure 1:**
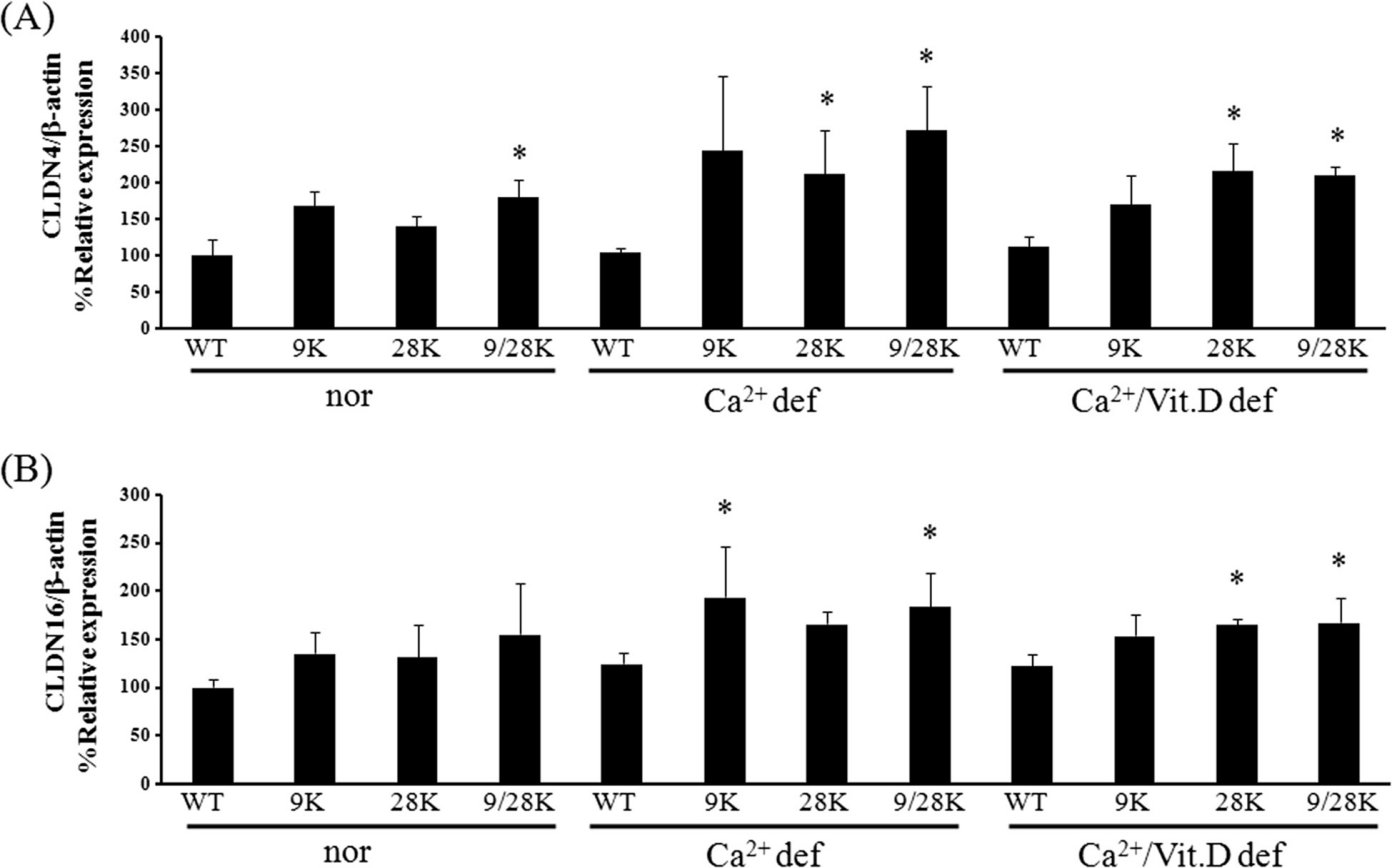
**Tissue-specific expression of tight junction mRNA in the kidney of mice.** CLDN4 **(A)** and CLDN16 **(B)** mRNA expression were analyzed by real-time PCR. The level of CLDN4, and CLDN16 mRNA of kidney in WT, CaBP-9 k KO, -28 k KO, and DKO animals was evaluated in normal or calcium deficiency diet or calcium/vitamin D-deficient. Every result was normalized relative to β-actin. The values represent means ± SD of three separate experiments. * indicates ^*^P < 0.05 *vs* WT of each diet.

In CaBP-28 k KO animals, CLDN4 mRNA expression was significantly up-regulated in the calcium-deficient (2-fold *vs* WT mice) and calcium/vitamin D-deficient (1.9-fold *vs* WT mice) groups, respectively (Figure 1A). CLDN4 mRNA expression was higher in DKO mice fed the normal (1.8-fold *vs* WT mice), calcium (2.6-fold *vs* WT mice), and calcium/vitamin D-deficient (1.9-fold *vs* WT mice) diet than those of WT mice. The level of CLDN4 expression was also higher in the calcium-deficient and calcium/vitamin D-deficient CaBP-28 k KO mice than ones fed the normal diet. While CLDN16 mRNA expression was not significantly altered by diet, it was higher in calcium-deficient CaBP-9 k KO (1.5-fold *vs* WT mice) and DKO mice (1.5-fold *vs* WT mice) as well as calcium/vitamin D-deficient CaBP-28 k KO (1.4-fold *vs* WT mice) and DKO mice (1.4-fold *vs* WT mice) compared to the WT animals (Figure 1B).

### Regulation of renal tight junction protein expression

**Figure 2:**
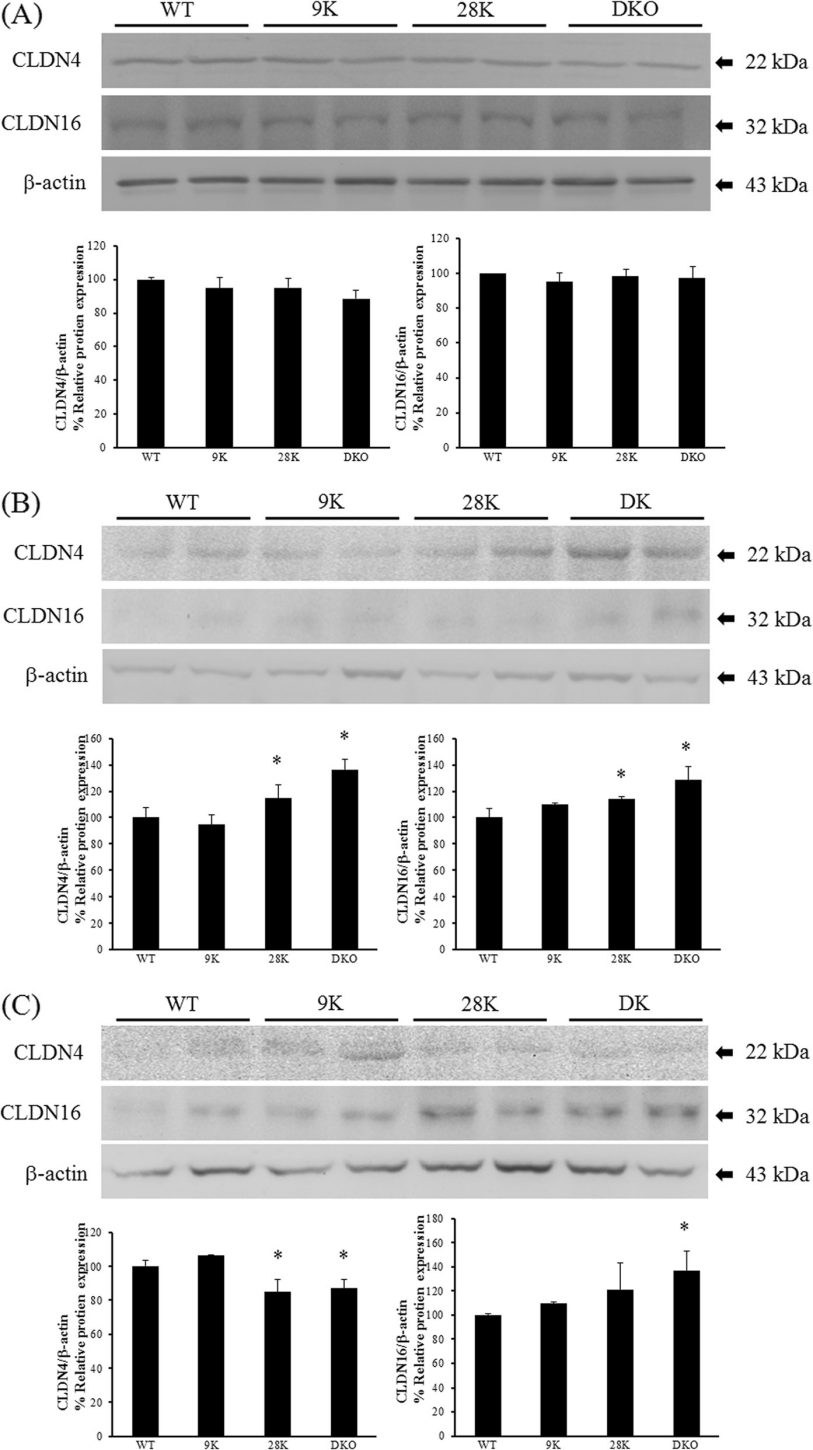
**Regulation of tight junction protein expression in the kidney.** The protein levels of CLDN4 and 16 were measured by Western blotting. The differential expression of CLDN4 and 16 in each type of normal conditioned mice were compared **(A)**. The impact of calcium deficiency on CLDN4, and CLDN16 protein expression in WT, CaBP-9 k KO, -28 k KO, and DKO animal was evaluated **(B)**. Differential CLDN4 and CLDN16 protein expression was examined in all strains of mice that were calcium/vitamin D-deficient **(C)**. β-actin was used as the control. The values represent means ± SD of three separate experiments. * indicates ^*^P < 0.05 *vs* WT of each diet.

The expression of CLDN4 and 16 proteins in the kidney was examined by Western blotting. Because the mRNA and protein expression patterns were similar, only the CLDNs showing significant induction of transcription level were chosen for Western blotting. In normal diet groups, CLDN4 and 16 protein expressions were not significantly changed (Figure 2A). In CaBP-28 k KO mice, CLDN4 protein levels appeared to be increased by the calcium- or calcium/vitamin D-deficient diet. With the calcium-deficient diet, CLDN4 protein level is increased in the CaBP-28 k KO and DKO mice (Figure 2B). CLDN16 expression appeared to be up-regulated in the DKO mice although the basal expression levels were low. In the calcium/vitamin D-deficient groups, CLDN4 and CLDN16 expression was up-regulated in the CaBP-28 k KO and DKO mice (Figure 2C). Overall, renal tight junction proteins were expressed in patterns similar to those of mRNA.

### Histologic alteration of renal tight junction proteins

**Figure 3:**
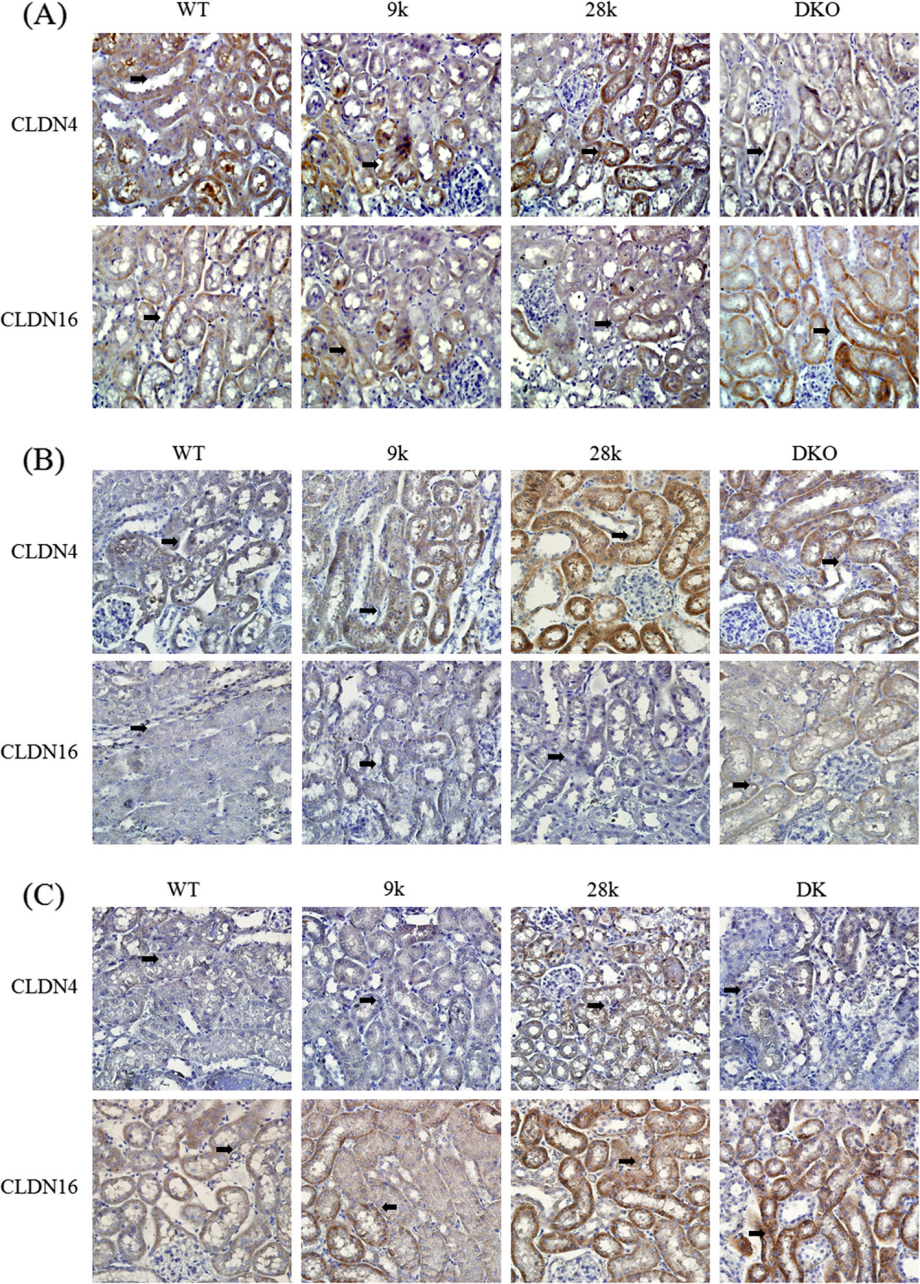
**Histologic alteration of tight junction proteins in the kidney.** Immunohistochemistry was used to evaluate the differential localization of CLDN4 and 16 in the kidney. In normal conditioned mice, the renal localization of CLDN4 and 16 were compared **(A)**. The effects of calcium deficiency on the differential localization of CLDN4 and CLDN16 were examined in the kidney of each strain of mice **(B)**. The localization of CLDN4 and 16 was evaluated in the kidney of calcium- and vitamin D-deficient WT, CaBP-28 k KO, and DKO mice **(C)**. Each slide was viewed at 400x magnification. Black arrows indicate immuno-positive signals.

Renal histologic alteration of CLDN4 and CLDN16 was examined by immunehistochemical analysis. CLDN4 and CLDN16 were found in all tubules of the kidney. Within the normal diet groups, there were no significant changes of the histologic differences of CLDN4, but CLDN16 were relatively higher in DKO mice (Figure 3A). CaBP-28 k KO mice fed the calcium-deficient and calcium/vitamin D-deficient diets showed strong immuno-positive signals specific for CLDN4. CLDN16 signals in CaBP-9 k KO mice that consumed the calcium-deficient diet were stronger than those found in WT mice, which is more enhanced in DKO mice (Figure 3B). Additionally, signals specific for CLDN4 was stronger in the CaBP-28 k KO and DKO mice than the WT animals. Changes in CLDN4 expression were most remarkable. With the calcium/vitamin D-deficient diet, CLDN4 and CLDN16 signals were much stronger in the CaBP-28 k KO and DKO mice compared to the WT counterparts (Figure 3C). Overall, changes in renal tight junction gene expression observed in this experiment appeared to correspond to the Western blot results, as shown as Figure 2.

## Discussion

Kidney is main regulator organ for the calcium homeostasis because it is only organ that calcium excretion and absorption are performed at the same time. Especially, regulating calcium reabsorption is most important for maintaining calcium homeostasis. If the kidney fails to maintain the calcium homeostasis, it leads of physiological disorders. Following calcium is reabsorbed from the renal tubule to blood through active transcellular and passive paracellular pathways [2], we therefore focused to investigating the two transports relationship in the kidney because it was not well studied. Therefore, the regulation of tight junction gene expression was evaluated in the kidney of mice lacking transcelluar proteins CaBP-9 k and/or CaBP-28 k. Furthermore, the animals were fed calcium deficient diet or calcium and vitamin D deficient diets to study the effects of dietary calcium intakes on tight junction gene expression.

According to earlier reports where the regulation of renal tight junction gene expression appeared in renal tubule, expression of common sealing transmembrane proteins (OCLN, CLDN1, and CLDN5) [27,28,32,33], renal cation channel-forming CLDNs (CLDN10b, CLDN16 and CLDN19) [21-24,26], cation barrier CLDN4 [29], and ZO-1 was examined in kidney of our null mouse models. Although in normal diet conditions, we did not observe significant differences in most of tight junction related transcripts of kidney in mice lacking CaBP-9 k or CaBP-28 k, the induction of renal CLDN4 transcripts in DKO mice were referred to as the evidence of link between transcellular transport and paracellular pathway. Moreover, mRNA expressions of several tight junction genes were significantly increased in calcium and calcium/vitamin D deficient diets according to genotypes including CaBP-9 k KO, CaBP-28 k KO or DKO mice but not WT mice. The increase of tight junction related transcripts are also another evidence explaining relation between transcellular transport and paracellular pathway because CaBP-9 k or CaBP-28 k are responsible for calcium transcellualr reabsorption in the kidney [3]. CLDN10b, CLDN16, and CLDN19 form cation channels in the TAL of Henle’s loop, and are critical for renal calcium reabsorption [34,35]. Among the calcium-related CLDNs in the kidney, CLDN10b appears to be the major compensatory regulator that may increase calcium reabsorption when the transcellular transport is impaired due to dietary calcium deficiency.

The histologic alteration of tight junction proteins was not observed in the TAL, the site of renal calcium reabsorption. Since paracellular pathway is bidirectional, up-regulation of these factors may reduce calcium ion leakage through the intercellular space in the renal nephrons and TAL. CLDN1 seals intercellular junctions, and is found in the glomerulus and proximal tubule [27,34], and its induction may be involved with the glomerular filtration rate. Levels of CLDN1 expression were higher in calcium-deficient CaBP-9 k KO and DKO mice compared to the WT counterparts. Our previous study of transcellular transport regulation with an experimental design similar to that of the current experiment has been conducted [36]. In previous study, transcellular calcium transport genes (TRPV5/6, NCX1, PMCA1b, and CaBP-9 k) were expressed in a compensatory manner in calcium-deficient and calcium/vitamin D-deficient CaBP-28 k KO and DKO mice. Taken together, the ablation of CaBPs could make not only the regulative expression of calcium transport genes, which belong to same transcellular transport, but also tight junction genes which belong to another.

CLDN14 has been reported as regulator of calcium transport in the kidney, and involved with calcium sensing receptor (CaSR) signaling via microRNA pathway [25]. Moreover, CLDN4 blocks calcium reabsorption in the TAL of kidney, by interacting with CLDN16 and 19 complexes [25]. When the transcript and protein of CLDN14 was investigated in present study (Additional file 1: Figure S1), CLDN14 mRNA was down-regulated in the calcium or calcium/vit.D deficient CaBP-9 k KO animals. However protein expression was not significantly changed in same groups. Histologic alteration pattern was similar with mRNA regulation except calcium/vit.D deficient CaBP-28 k KO group. In calcium or calcium/vit.D deficient condition, reduction of renal CLDN14 in CaBP-9 k KO mice may be involved with renal calcium reabsorption compensating to deficient calcium absorption due to CaBP-9 k KO animals in calcium or calcium/vit.D deficient condition [37].

The absence of CaBP-28 k in kidney does not appear to directly impact tight junction expression because the relative expressions of tight junction protein in CaBP-28 K KO mice were no longer observed in normal diet condition. However, in calcium-deficient and calcium/vitamin D-deficient environment, the limitation of calcium and vitamin D absorption induced the expression of tight junction protein in CaBP-28 k and DKO mice limiting transcellular transport.

## Conclusions

In summary, our results suggest that transcellular and paracellular pathways are functionally cooperating for calcium reabsorption in the kidney, and they could be alternative to each other.

## Abbreviations

APS: Amino silane; BBB: Blood–brain barrier; CaBP-9 k: Calbindin-D_9k_; CaBP-28 k: Calbindin-D_28k_; cDNA: Complementary DNA; CLDN: Claudin; CT: Theshold cycle; FHHNC: Familial hypomagnesaemia with hypercalciuria and nephrocalcinosis; kDa: kilodalton; KO: Knock-out; M-MLV: Moloney murine leukemia virus; OCLN: Occludin; PBS: Phosphate buffer saline; PDZ: PSD95/Dlg/ZO-1; RT: Reverse transcription; sv40: Simian virus 40; SDS-PAGE: Sodium dodecyl sulfate polyacrlamide gel electrophoresis; TAL: Thick ascending limb of Henle’s loop; TBS-T: Tris-buffered salined tween-20; WT: Wild-type; ZO: Zona occludens.

## Competing interests

The authors declare that they have no competing interests.

## Authors’ contributions

IH designed and carried out most of experiments and wrote manuscript. HY, HSK and CA performed all *in vivo* experiments such as qRT-PCR, Western blotting and immunohistochemistry. EJH and BSA refined experimental design or protocols, and performed proofreading of manuscript. EBJ proposed the experiments, directed all the experiments, and wrote the manuscript. All authors were involved in interpreting data, and editing manuscripts together. The final manuscript was read and approved by all authors.

## Authors’ information

Prof. Eui-Bae Jeung, D.V.M., PhD, Laboratory of Veterinary Biochemistry and Molecular Biology, College of Veterinary Medicine, Chungbuk National University, Cheongju, Chungbuk, 361–763, Republic of Korea; Phone: +82-43-261-2397; Fax: +82-43-267-3150; Email: ebjeung@chungbuk.ac.kr.

## Supplementary Material

Additional file 1: Figure S1 Renal CLDN14 mRNA expression of mice. The mRNA expression of CLDN14 in the kidney of WT, CaBP-9k, CaBP-28k, and DKO were analyzed by real-time PCR. * indicates *P < 0.05 *vs* WT of each diet. Click here for file
