# Supplementary material for: Regulation of tight junction gene expression in the kidney of calbindin-D9k and/or -D28k knockout mice after consumption of a calcium- or a calcium/vitamin D-deficient diet
Source: BMC Biochem. 2014 Mar 15;15:6. doi: 10.1186/1471-2091-15-6 (PMC4003857; doi:10.1186/1471-2091-15-6)
Supplement: Additional file 1: Figure S1 — Renal CLDN14 mRNA expression of mice. The mRNA expression of CLDN14 in the kidney of WT, CaBP-9k, CaBP-28k, and DKO were analyzed by real-time PCR. * indicates *P < 0.05 vs WT of each diet. [file 1471-2091-15-6-S1.docx]

**Additional file 1: Figure S1**

**
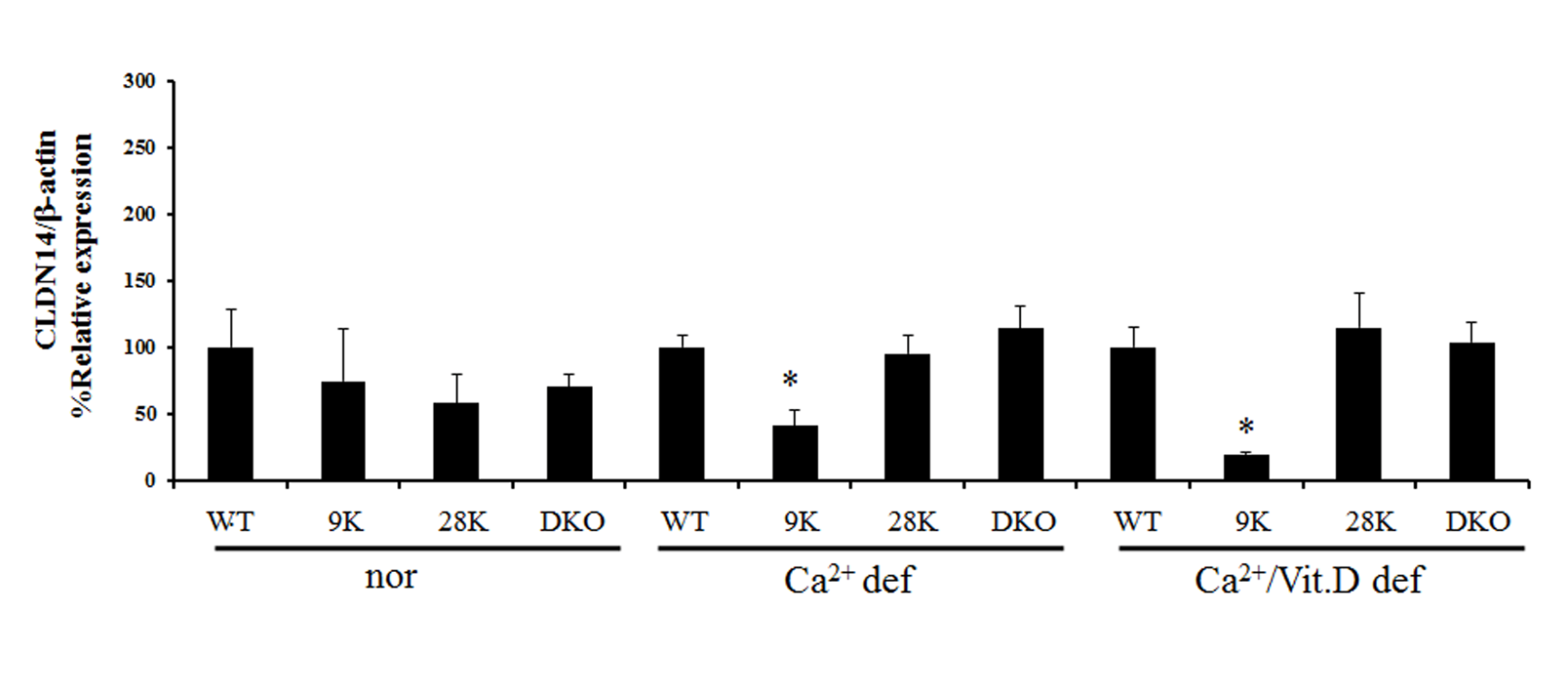
**

**Additional file 1: Figure 1. Renal CLDN14 mRNA expression of mice.**

The mRNA expression of CLDN14 in the kidney of WT, CaBP-9k, CaBP-28k, and DKO were analyzed by real-time PCR. * indicates *^*^P*<0.05 *vs* WT of each diet

**Additional file 2: Figure S2**


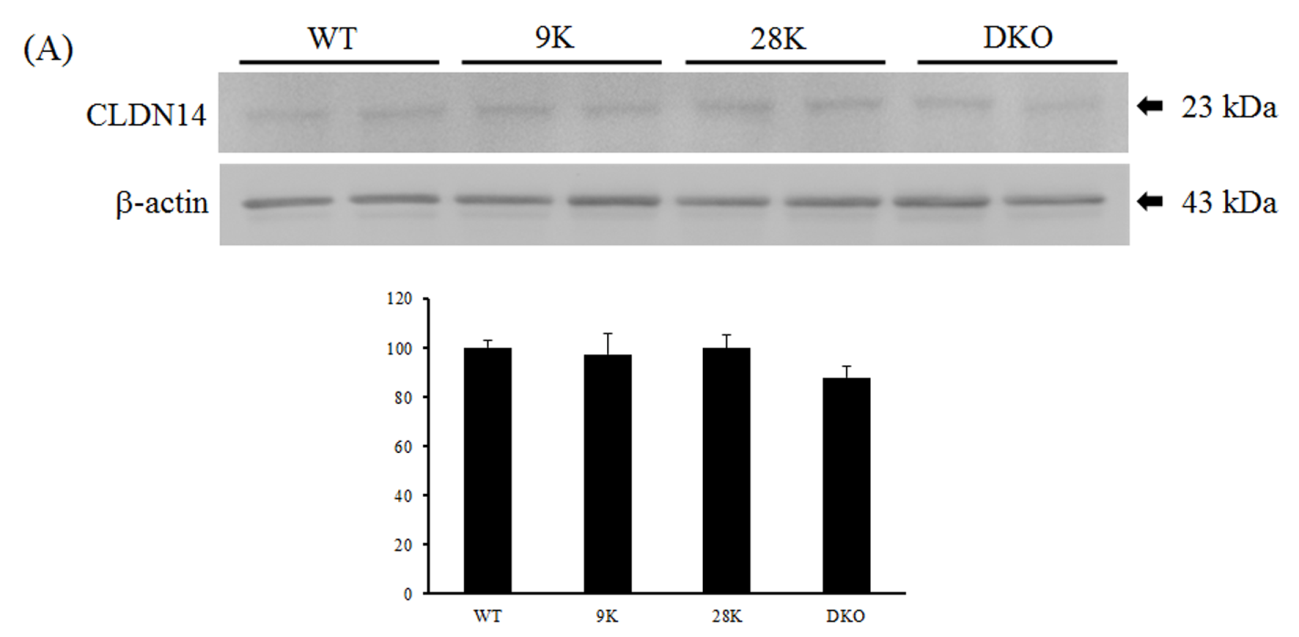

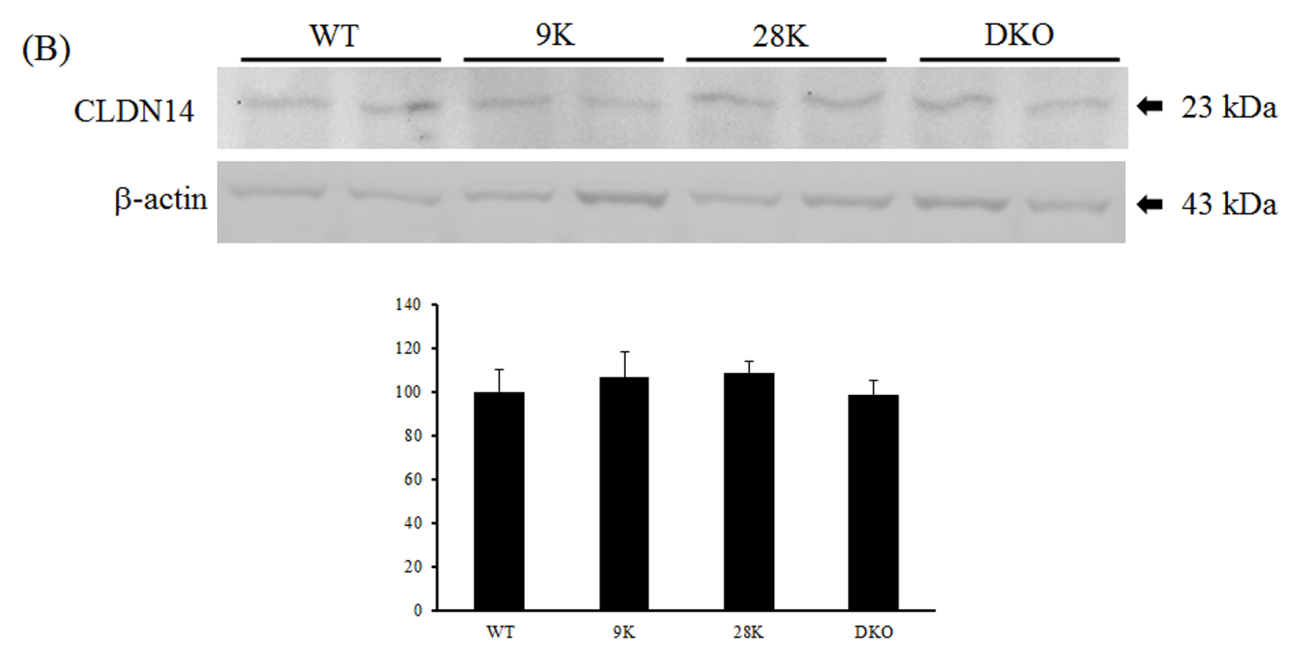


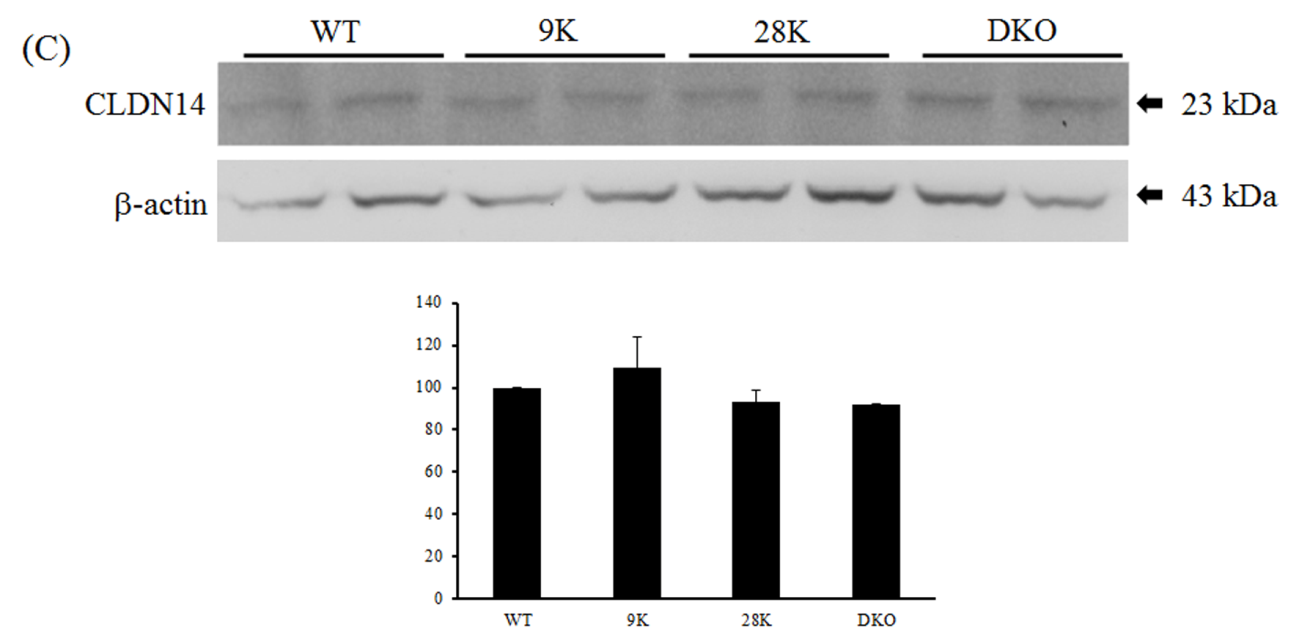


**Additional file 2: Figure 2. CLDN14 protein expression in the kidney of mice.**

The expression level of CLDN14 protein was measured by western blotting in normal (A), calcium deficient (B) and calcium/vit.D deficient diet condition (C).

**Additional file 3: Figure S3**


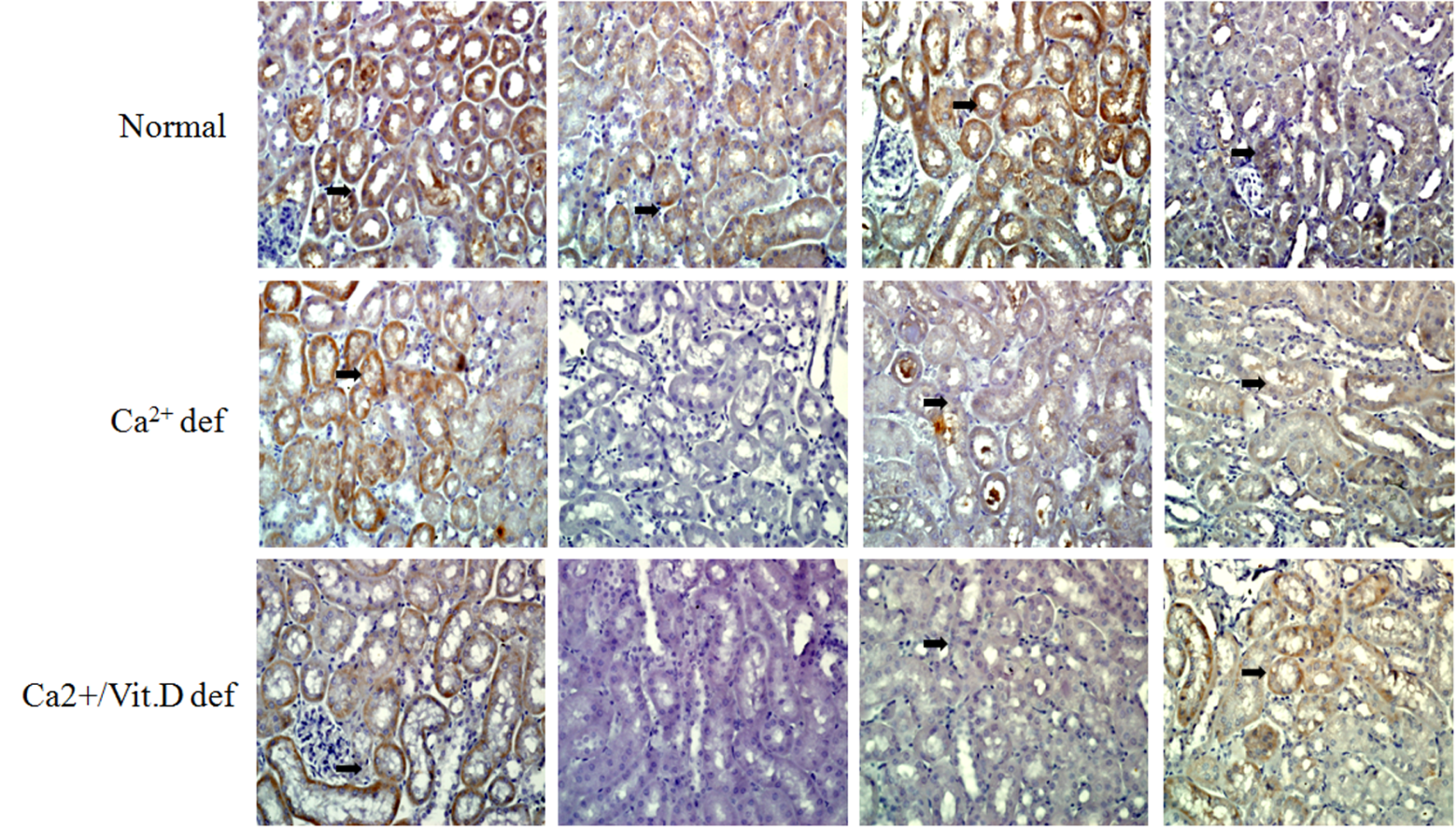


**Additional file 3: Figure 3. Histologic alteration of CLDN14 in the kidney of mice.**

The histologic alteration of CLDN14 in the kidney was evaluated by immunohistochemistry. The differential renal CLDN14 localization of WT, CaBP-9k KO, CaBP-28k KO and DKO mice were compared in normal, calcium deficient and calcium/vit.D deficient condition. Each slide was viewed at 400x magnification. Black arrows indicate immuno-positive signals.
